# Novel Antimicrobial Strategies to Prevent Biofilm Infections in Catheters after Radical Cystectomy: A Pilot Study

**DOI:** 10.3390/life12060802

**Published:** 2022-05-27

**Authors:** Rosa Gaglione, Katia Pane, Maria De Luca, Monica Franzese, Angela Arciello, Francesco Trama, Stefano Brancorsini, Marco Salvatore, Ester Illiano, Elisabetta Costantini

**Affiliations:** 1 Department of Chemical Sciences, University of Naples Federico II, 80126 Naples, Italy; rosa.gaglione@unina.it (R.G.); maria.deluca2@unina.it (M.D.L.); anarciel@unina.it (A.A.); 2 Istituto Nazionale di Biostrutture e Biosistemi (INBB), 00136 Rome, Italy; 3 IRCCS Synlab SDN, Via E. Gianturco 113, 80143 Naples, Italy; monica.franzese@synlab.it (M.F.); direzionescientifica.irccssdn@synlab.it (M.S.); 4 Andrological and Urogynecological Clinic, Santa Maria Terni Hospital, University of Perugia, 05100 Terni, Italy; francescotrama@gmail.com (F.T.); ester.illiano@inwind.it (E.I.); elisabetta.costantini@unipg.it (E.C.); 5 Department of Experimental Medicine, University of Perugia, 06132 Perugia, Italy; stefano.brancorsini@unipg.it

**Keywords:** microbial communities, urinary catheter-associated infections, radical cystectomy, antimicrobial peptides, antibiofilm agents, conventional antibiotics, antimicrobial resistance, combination therapy

## Abstract

Catheter-associated infections in bladder cancer patients, following radical cystectomy or ureterocutaneostomy, are very frequent, and the development of antibiotic resistance poses great challenges for treating biofilm-based infections. Here, we characterized bacterial communities from catheters of patients who had undergone radical cystectomy for muscle-invasive bladder cancer. We evaluated the efficacy of conventional antibiotics, alone or combined with the human ApoB-derived antimicrobial peptide r(P)ApoB_L_^Ala^, to treat ureteral catheter-colonizing bacterial communities on clinically isolated bacteria. Microbial communities adhering to indwelling catheters were collected during the patients’ regular catheter change schedules (28 days) and extracted within 48 h. Living bacteria were characterized using selective media and biochemical assays. Biofilm growth and novel antimicrobial strategies were analyzed using confocal laser scanning microscopy. Statistical analyses confirmed the relevance of the biofilm reduction induced by conventional antibiotics (fosfomycin, ceftriaxone, ciprofloxacin, gentamicin, and tetracycline) and a well-characterized human antimicrobial peptide r(P)ApoB_L_^Ala^ (1:20 ratio, respectively). Catheters showed polymicrobial communities, with Enterobactericiae and Proteus isolates predominating. In all samples, we recorded a meaningful reduction in biofilms, in both biomass and thickness, upon treatment with the antimicrobial peptide r(P)ApoB_L_^Ala^ in combination with low concentrations of conventional antibiotics. The results suggest that combinations of conventional antibiotics and human antimicrobial peptides might synergistically counteract biofilm growth on ureteral catheters, suggesting novel avenues for preventing catheter-associated infections in patients who have undergone radical cystectomy and ureterocutaneostomy.

## 1. Introduction

Bladder cancer affects mainly males, with ≈70% of diagnoses being non-muscle-invasive bladder cancer (NMBICs) and the rest being muscle-invasive disease (MBICs) [1,2,3]. Radical cystectomy and ureterocutaneostomy, are recommended for advanced (>T2) muscle-invasive bladder cancer. However, complications such as long-term catheter use is the major risk factor for developing a catheter-associated urinary tract infection (CAUTI) in these patients [4,5]. CAUTIs for long-term catheterization are associated with increased morbidity and mortality and generally are caused by polymicrobial bacterial and fungi communities [1,6,7]. The commonly isolated bacterial uropathogens belong to the Enterobactericiae such as Enterobacter, Escherichia, and Klebsiella, e.g., *Klebsiella pneumoniae*, as well as Proteus species, but they can be also caused by Gram-positive bacteria including Staphylococcus species [1,6].

Currently, the value of antibiotic prophylaxis at catheter removal is unclear [4,8,9]. In catheterized patients, cloudy urine, fever, and urease bacteria (e.g., *Proteus mirabilis*) are major symptoms of a potential CAUTI [1]. These conventionally are defined as bacterial growth >10^3^ CFU/mL in a single catheter urine specimen or a midstream voided-urine specimen within 48 h of catheter removal [6,10].

Indeed, long-term catheter use may perturb the balance between the host immune defense and the microbiome of the UT epithelium [1], leading to infections [11]. Bacterial biofilms adherent to urinary catheters may include pathogenic and multi-drug-resistant species, which are difficult to treat with antibiotics [1,10,12].

Therefore, novel antimicrobial strategies and cutting-edge technologies are needed to fight biofilm persistence on biomedical devices. Natural and rationally designed antimicrobial peptides (AMPs) might be promising candidates, less prone to developing multi-drug-resistant strains [13,14]. AMPs represent an ancient host defense weapon of all living organisms against pathogens [13,14,15]. They are endowed with antimicrobial, antibiofilm, immunomodulatory, antitumor, and wound healing activities [13,14,15]. AMPs are well suited to fight biofilm-formed infections due to their ability to penetrate and destroy the complex biofilm structure [16]. Indeed, combining antibiotics and antimicrobial peptides might represent an effective strategy against broad-spectrum bacterial infections [17,18]. Several studies have highlighted the synergy between membranolytic antimicrobial peptides and conventional antibiotics [17,18,19,20]. We have previously characterized the human ApoB-derived antimicrobial peptide’s multifunctional activities (ApoB, residues 887–922), named r(P)ApoB_L_^Ala^ [21]. This peptide has antimicrobial and antibiofilm properties and showed synergistic effects in combination with ciprofloxacin, even against clinically isolated bacteria from cystic fibrosis patients [20,21,22,23,24,25,26,27]. Worthy of note, the recombinant peptide does not exert toxicity effects on human cell lines, opening the way to many potential biomedical applications.

Here, we assessed the antimicrobial and antibiofilm susceptibility to conventional antibiotics, alone or combined with the human ApoB-derived antimicrobial peptide r(P)ApoB_L_^Ala^, on clinically isolated bacteria from ureteral catheters of patients who have undergone radical cystectomy for muscle-invasive bladder cancer. We found as predominent Gram-negative species the *Enterobactericiae*, and *Staphylococcus* as Gram-positive strains. r(P)ApoB_L_^Ala^ showed antimicrobial efficacy against the planktonic and the biofilm-forming communities of each clinically isolated bacteria visualized by using confocal laser scanning microscopy (CLSM). To several extents, r(P)ApoB_L_^Ala^ demonstrated synergizing with ceftriaxone and fosfomycin, two conventionally used antibiotics in urological settings to reduce biofilm structure. This study shows preliminary data for the development of new antimicrobial strategies for the prevention and treatment options of uropathogens in the age of antibiotic resistance.

## 2. Materials and Methods

### 2.1. Materials

Patients were fitted with polyvinyl chloride (PVC) ureteral catheters (Coloplast Group, Holtedam 1, Humlebaek, DK 3050, Denmark). Extracted catheters were used for biofilm analysis. Half the patients had 9 ch diameter and half had 10 ch diameter catheters, according to the diameter of the ureter. Confocal laser scanning microscopy analyses in static conditions were performed using Thermo Scientific™ Nunc™ Lab-Tek™ Chambered Coverglass systems (Thermo Fisher Scientific, Waltham, MA, USA).

### 2.2. Reagents

The antibiotics were fosfomycin, ceftriaxone, ciprofloxacin, gentamicin, and tetracycline. The differential/selective media and reagents employed for biochemical tests (urea-broth, simmon citrate agar, Hi-Crome UTI agar, cetrimide agar, methyl red (MR) test, Voges–Proskauer (VP) test, indole spot reagent, Ureasi test, and spot indole test) were purchased from Sigma-Merck (Milan, Italy), unless otherwise specified. FilmTracer™ SYPRO™ Ruby Biofilm Matrix Stain was purchased from Thermo Fisher Scientific (Waltham, MA, USA).

### 2.3. Recombinant Peptide Production

Recombinant ApoB-derived peptide was expressed and isolated as previously described [22,28].

### 2.4. Extraction of Biological Samples from Catheters

Biological samples were extracted from the catheters, cut into 2 cm pieces, and immediately transferred into 5 mL sterile PBS in 50 mL tubes. Samples were vortexed for 1 min, sonicated for 5 min in an ultrasonic bath, and vortexed for a further minute. Serial dilutions were plated on selective and differential media.

### 2.5. Antimicrobial Activity Assays

The antimicrobial activity of r(P)ApoB_L_^Ala^ in combination with antibiotics was evaluated as previously described [21]. Briefly, bacterial samples were grown to mid-logarithmic phase in MHB at 37 °C and then diluted to 4 × 106 CFU/mL in Difco 0.5X Nutrient Broth (NB, Becton-Dickenson, Franklin Lakes, NJ, USA) and mixed 1:1 *v*/*v* with twofold serial dilutions of antibiotics (Table 1). Following overnight incubation, MIC100 values were determined as the lowest antibiotic concentration responsible for no visible bacterial growth. All experiments were carried out in three independent replicates.

The minimal inhibitory concentration (MIC100) was determined as the lowest concentration of compound/mixture resulting in no bacterial growth.

### 2.6. Analysis of Antibiofilm Activity by Confocal Laser Scanning Microscopy (CLSM)

The antibiofilm activity of the antimicrobial peptide r(P)ApoB_L_^Ala^ in combination with antibiotics was evaluated by CLSM as previously reported in [21,29]. Samples were stained with FilmTracer™ SYPRO™ Ruby Biofilm Matrix Stain (Thermo Fisher Scientific, Waltham, MA, USA).

### 2.7. Statistical Analyses

Statistical analyses were performed by R version 3.6.3 (29 February 2020) [30], dplyr [31], rstatix [32], and ggplot2 [33] packages. For CLSM fluorescence intensity, considering the skewed distribution for each variable, we applied non-parametric tests on CLSM data upon inspection of normality. We used the Kruskal–Wallis test for global *p*-values and the Wilcoxon test for pairwise comparisons of CLSM data, with “Untreated” biofilm, or single agent as a comparison group. A *p*-value of <0.05 was considered statistically significant. CLSM Z-stack images across each acquisition layer were compared by multiple comparison Fisher’s exact test, FDR method, with a statistical significance threshold ≤0.05 by RVAideMemoire v 0.9–79 package [34]. For CLSM thickness, MultCompView version 0.1–8 R packages were used to calculate one-way ANOVA and Tukey’s HSD post hoc tests for all group comparisons, with default p-adjusted correction.

## 3. Results

### 3.1. Catheter Collection, Microbial Strain Extraction, and Assessment

We evaluated novel antimicrobial strategies against bacterial planktonic and adherent communities extracted from six ureteral catheters during patient-scheduled catheter exchange. Catheters were changed after 28 days, except for patient U5, who changed the catheter after 20 days due to probe obstruction. Detailed data are reported in Table S1.

**Figure 1:**
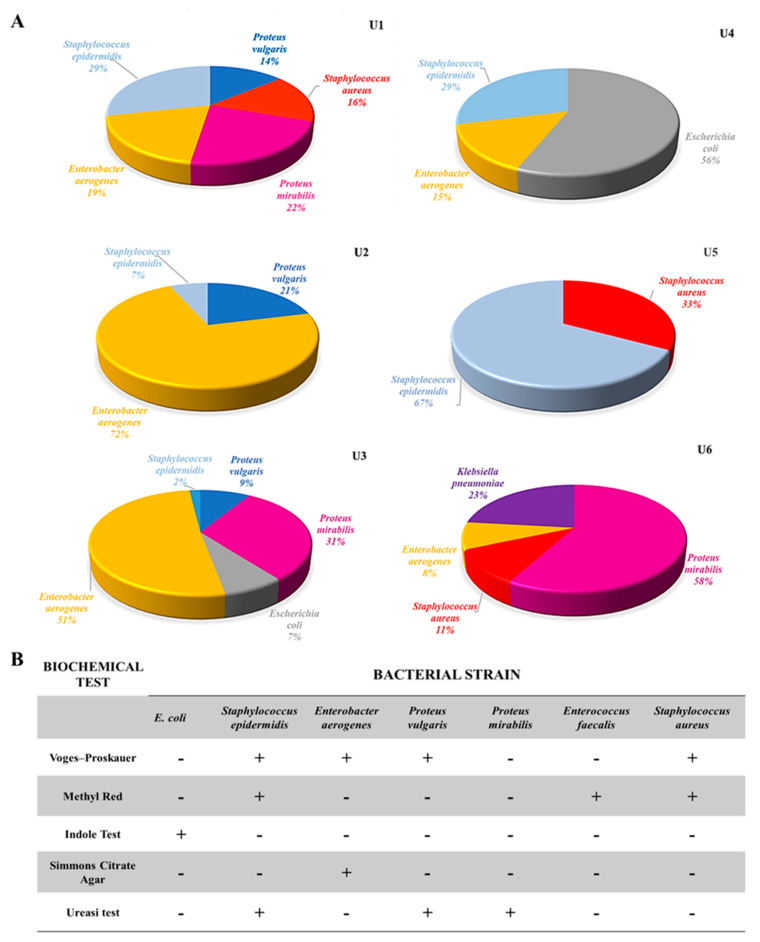
Analysis of the heterogeneous composition of microbial communities extracted from patients’ ureteral catheters. (**A**) Pie charts showing the bacterial composition of heterogeneous microbial communities extracted from ureteral catheters for each patient. (**B**) Identification of isolated bacterial strains according to biochemical tests.

To isolate, identify, and characterize bacterial strains from the catheters, serial dilutions of each extract were plated on selective and differential media. We found a clear predominance of Gram-negative bacteria; the most frequently isolated strains were *Enterobacter aerogenes* and *Proteus mirabilis*, followed by the Gram-positive bacteria *Staphylococcus epidermidis* (Figure 1A). The heterogeneous bacterial composition for each extract was consistent with previous findings. Bacterial strains were identified using well-established biochemical assays (Figure 1B).

### 3.2. Evaluation of Antimicrobial Activity towards Isolated Bacterial Communities

The antimicrobial activity of antibiotics against the microbial communities extracted from the catheters was evaluated using the broth microdilution method [21]. As expected, antibiotics inhibited the planktonic microbial growth at different MIC concentrations. Fosfomycin was effective at a very low concentration range (0.15–0.3 mg/mL) compared with ceftriaxone, tetracycline, gentamycin, and ciprofloxacin (Table 1). The latter reached MIC_90_ inhibition in all cases at 0.625 mg/mL. Interestingly, the MIC value for the antimicrobial peptide r(P)ApoB_L_^Ala^ was >0.4 mg/M in all cases.

### 3.3. Evaluation of Antimicrobial Activity by CLSM

**Figure 2:**
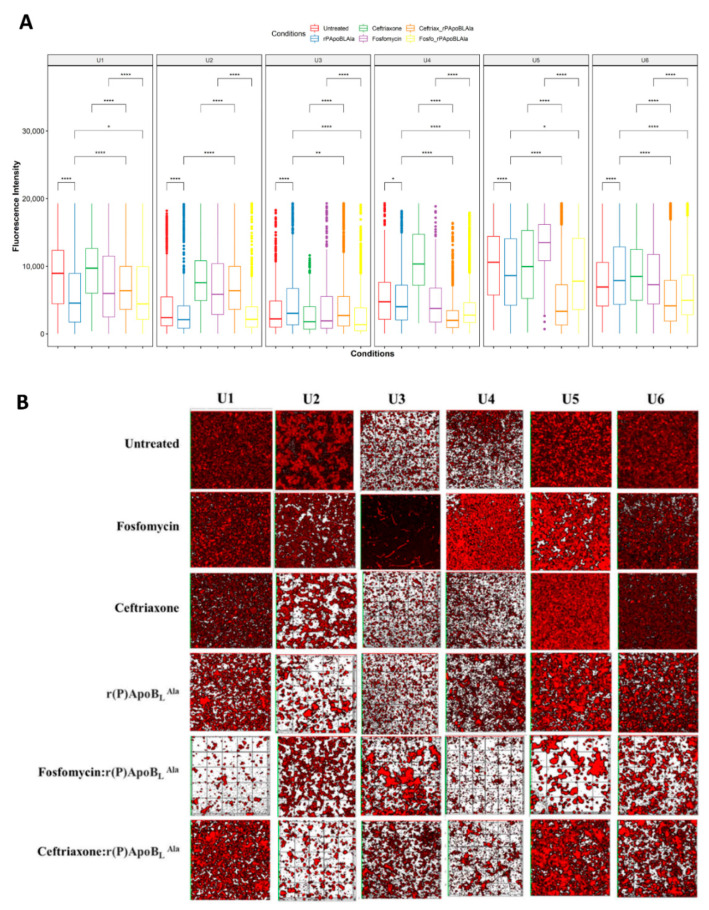
Analysis of the antibiofilm activity of the antimicrobial peptide r(P)ApoB_L_^Ala^ and of the antibiotics fosfomycin or ceftriaxone determined by confocal laser scanning microscopy (CLSM) in static live conditions. Agents were tested alone or in combination. (**A**) Fluorescence intensities determined by CLSM after 24 h in the absence (control group) or in the presence of r(P)ApoB_L_^Ala^ of the antibiotics fosfomycin or ceftriaxone, or of combinations of the antimicrobial peptide with each antibiotic. Statistical significance codes are shown only for the significant differences in the comparison of interest as * *p* < 0.05, ** *p* < 0.01, **** *p* < 0.0001. (Pairwise Wilcoxon tests, adjusted *p*-value according to the Benjamini–Hochberg method.) (**B**) Three-dimensional reconstructions of biofilm images acquired by CLSM for each catheter extract in the absence or in the presence of antibiofilm agents under study.

We previously demonstrated that r(P)ApoB_L_^Ala^ peptide can combat biofilm-associated bacterial infections in cystic fibrosis, either alone or conventional antibiotics [24]. Therefore, we investigated their effectiveness in countering catheter-associated biofilm infections using CLSM experiments in static conditions. The extracted samples were grown in static conditions for 24 h and stained using FilmTracer™ SYPRO™ Ruby Biofilm Matrix Stain (Figure 2).

In each case, we verified that the 3D reconstructions of the z-stack images (lower, middle, and upper) acquired by CLSM were representative of the whole channel for each condition (Table S2). Comparing biofilm growth after 24 h in untreated versus treated conditions, we observed fluorescence mean intensity changes (arbitrary units, a.u.) for the six patients’ extracts (*p* < 0.05, Figure S1), indicating significant alterations in biofilm morphology compared to the control, except for fosfomycin alone in U3 and ceftriaxone alone in U5 treatment compared with the untreated samples, respectively (*p* > 0.05, Figure S1).

To exploit any potential synergistic effects between the r(P)ApoB_L_^Ala^ peptide and conventional antibiotics, we analyzed biofilm growth upon treatment with r(P)ApoB_L_^Ala^ peptide alone, antibiotics alone, and combinations. As previously described, the measurements recorded for each condition are representative of the whole channel (Table S3). We compared the effects of r(P)ApoB_L_^Ala^ plus ceftriaxone, or r(P)ApoB_L_^Ala^ plus fosfomycin in 1:20 ratio combinations with those of the single agents at the same concentrations. In each extract, we observed biofilm changes from dispersed to aggregated patches in the presence of r(P)ApoB_L_^Ala^ peptide alone or in combination (Figure 2A,B). Ceftriaxone plus r(P)ApoB_L_^Ala^ combinations and fosfomycin plus r(P)ApoB_L_^Ala^ combinations reduced biofilm coverage in U1, U2, U4, U5, and U6 extracts in a statistically significant manner, compared with peptide or antibiotic alone (*p* < 0.01, Figure 2A).

We further investigated synergistic effects to further investigate the r(P)ApoB_L_^Ala^ antimicrobial effectiveness. We assessed the antibiofilm effects of several antibiotics such as ciprofloxacin, gentamicin, and tetracycline alone or in combination with r(P)ApoB_L_^Ala^ in U1, U3, and U5 extracts. As before, statistically significant reductions in the biofilm were mediated by each antimicrobial agent, alone or in combination, compared with untreated biofilm (*p* < 0.05, Figure S2), except for ciprofloxacin treatment of the U3 extract (*p* > 0.05, Figure S2).

**Figure 3:**
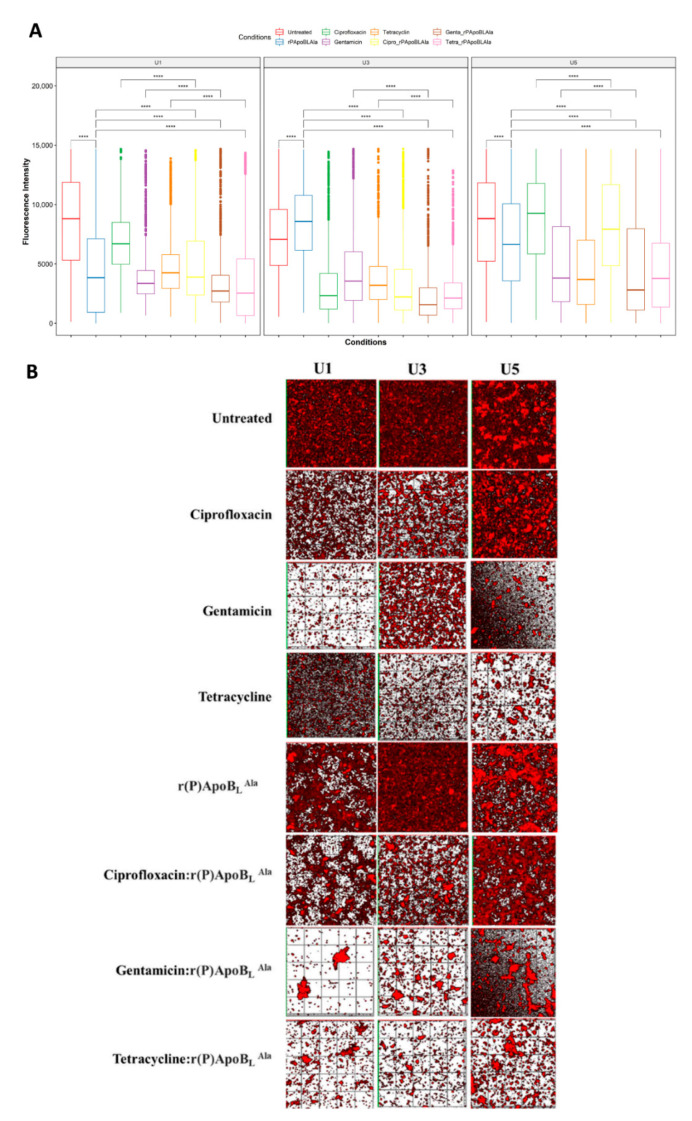
Analysis of the antibiofilm activity of the antimicrobial peptide r(P)ApoB_L_^Ala^ and the antibiotics ciprofloxacin, gentamicin, or tetracycline by confocal laser scanning microscopy (CLSM) in static live conditions. Agents were tested alone or in combination. (**A**) Fluorescence mean intensities determined by CLSM upon 24 h in the absence (control group) or in the presence of r(P)ApoB_L_^Ala^, of the antibiotics ciprofloxacin, gentamicin, or tetracycline, or of combinations of the antimicrobial peptide with each antibiotic. Statistical significance codes are shown only for the significant differences in the comparison of interest as **** *p* < 0.0001. (Pairwise Wilcoxon tests, adjusted *p*-value according to the Benjamini–Hochberg method.) (**B**) Three-dimensional reconstructions of biofilm images acquired by CLSM for each catheter extract in the absence or in the presence of antibiofilm agents under study.

When we measured the antibiofilm effects of the combinations compared with the single agents by CLSM image analyses (Figure 3), we observed a huge biofilm dispersion mediated by combinations of r(P)ApoB_L_^Ala^ plus gentamicin or r(P)ApoB_L_^Ala^ plus tetracycline.

**Figure 4:**
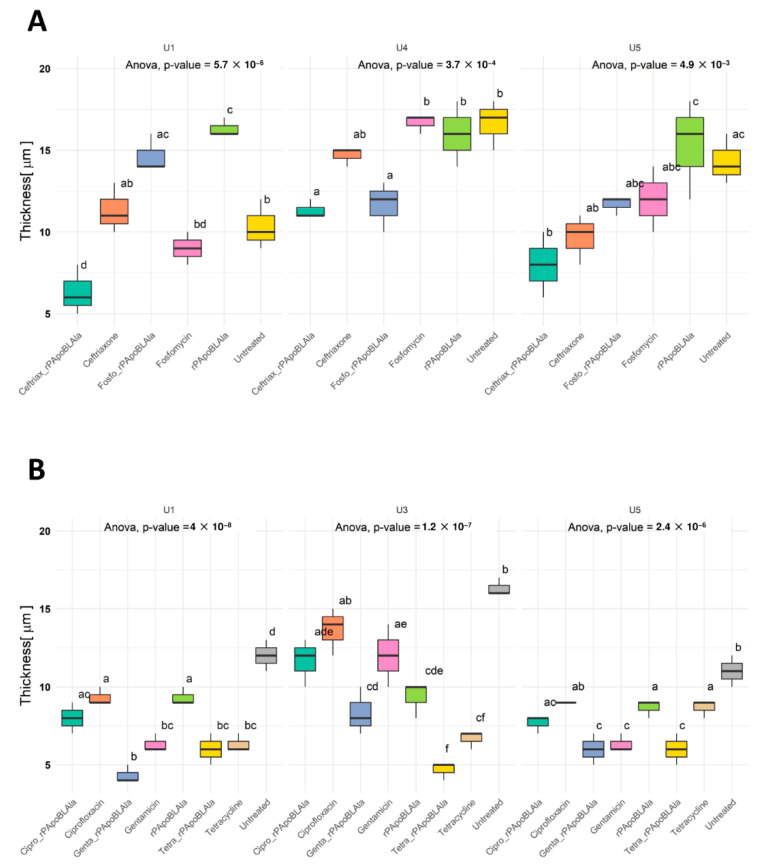
Average biofilm thickness by confocal laser scanning microscopy (CLSM). (**A**) Thickness of individual-patient-derived catheter extracts U1, U4, and U5 upon incubation with antimicrobial agents r(P)ApoB_L_^Ala^ fosfomycin or ceftriaxone alone or in combination with peptide. (**B**) Thickness of individual-patient-derived catheter extracts U1, U3, and U5 upon incubation with antimicrobial agents r(P)ApoB_L_^Ala^ or ciprofloxacin, gentamicin, or tetracycline alone or in combination with peptide. One-way ANOVA of the thickness was determined independently. Letter-based plot (a–f letters) for statistical Tukey’s multiple comparison post hoc test significance. Boxes with no letters in common are significantly different (*p* adjusted *<* 0.05).

Indeed, in clinical bacterial isolates from U1 and U3 catheters, both these combinations were significantly more effective than treatment with either peptide alone or antibiotics alone (*p* < 0.05). Only in the case of the U5 extract was the combination of r(P)ApoB_L_^Ala^ and tetracycline less effective than the single antibiotic agent (*p* > 0.05, Figure 3A). In addition, ciprofloxacin alone or in combination did not affect biofilm growth in the U3 extract (Figure 3). We also analyzed biofilm thickness to evaluate the reduction in biofilm height after 24 h of incubation with the single agent or with the combinations. For each extract, biofilm thickness in untreated samples ranged from 9.00 ± 1.00 (mean ± SD, U6 extract) to 17.33 ± 3.06 μm (mean ± SD, U4 extract, Figure 4). Statistically significant differences in terms of average thickness reduction were present in U1 (*p*-value < 0.01), U4 (*p*-value = 0.00037), and U5 extracts (*p*-value = 0.0049), according to one-way ANOVA analysis (Figure 4A).

Pairwise comparison between all conditions showed that r(P)ApoB_L_^Ala^ plus ceftriaxone exerted the most robust biofilm height reduction for each extract (Figure 4A, boxes with no letters in common were significantly different). However, in terms of synergistic effects, we found that, for the U1 extract, the combination r(P)ApoB_L_^Ala^ plus ceftriaxone was significantly different compared to the treatment with the single antibiotic agent or with the peptide alone (*p* < 0.05, Figure 4A and Table S4). Similarly, for the U4 extract, we observed statistically significant differences when comparing the combination of r(P)ApoB_L_^Ala^ plus fosfomycin compared to fosfomycin alone or peptide alone (*p*-adjusted < 0.05, Figure 4A and Table S4).

These results for U1, U3, and U5 extracts in the presence of ciprofloxacin, gentamicin, or tetracycline (alone or in combination with r(P)ApoB_L_^Ala^) indicated a significant biofilm reduction, i.e., U1 (*p*-value = 4 × 10^−8^), U3 (*p*-value = 1.2 × 10^−7^), and U5 (*p*-value = 2.4 × 10^−6^). Moreover, the observed biofilm height reduction reflected the 3D image reconstructions (Figure 3); indeed, the most effective treatment was gentamicin or tetracycline in combination with the peptide (Figure 4B). We observed potential synergistic effects and statistically significant differences in the average biofilm thickness in the U3 extract by comparing r(P)ApoB_L_^Ala^ plus gentamicin with a single antibiotic or peptide. Similarly, in the case of the U5 extract, by comparing r(P)ApoB_L_^Ala^ plus tetracycline with the single agents, we found statistically significant differences in average biofilm thickness (i.e., compared with peptide or antibiotic alone, *p*-adjusted < 0.05, Table S5).

## 4. Discussion

Catheter-associated UTIs are the leading cause of secondary healthcare-associated bacteraemia, representing ≈20% of hospital-acquired bacteremias [1,4,6]. Radical cystectomy is the gold-standard treatment for patients with MIBC or with NMIBC who fail intravesical treatment [2,4]. However, long-term catheterization increases their risk of complications [5,6,7]. In an attempt to assess the antimicrobial efficacy of novel antimicrobial strategies, we analyzed six patient catheter extracts and the effects of the human antimicrobial peptide, i.e., r(P)ApoB_L_^Ala^ [20,21,22,23,24,25,26,27]. We characterized the composition of catheter-extracted planktonic and adherent communities and evaluated their susceptibility to conventionally prescribed antibiotics, either alone or in combination with the human antimicrobial peptide. The 28-day ureteral catheter extracts were polymicrobial, with Gram-positive and Gram-negative species, indicated by broth microdilution and biochemical assays. We found *Enterobacter aerogens, Escherichia coli, Klebsiella pneumoniae, Staphylococcus aureus,* and *Proteus mirabilis* bacteria. Although antibiotics are valuable tools for preventing and curing infections, prudence in their use is necessary to prevent antibiotic resistance. Here, we assessed the antimicrobial efficacy of most of the antibiotics recommended by EAU guidelines for UTIs [4,10] (fosfomycin and ceftriaxone) and some less common antibiotics (ciprofloxacin, gentamicin, tetracycline) against microbial communities from each patient.

We investigated the biofilm-growing capacity of each extract in the presence of either conventional antibiotics or the human antimicrobial peptide r(P)ApoB_L_^Ala^. We tested at fixed ratios combinations of both antibiotic and peptide. We used confocal laser scanning microscopy (CLSM) to assess biofilm depletion in living conditions. Fluorescence measurements of biofilm morphology showed that conventionally prescribed fosfomycin and ceftriaxone significantly affect biofilm growth, although they were not equally effective on all patient extracts tested. The antimicrobial peptide r(P)ApoB_L_^Ala^ treatment reduced biofilm thickness and coverage. Investigating potential synergistic effects between r(P)ApoB_L_^Ala^ and antibiotics (1:20, mol/mol ratio), we found that combinations of ceftriaxone or fosfomycin with r(P)ApoB_L_^Ala^ were more effective than single agents (*p* < 0.01) (except for samples from patient U3). We also found that conventional antibiotic classes (gentamicin, ciprofloxacin, and tetracycline), alone or in combination with the peptide, effectively reduced growth of biofilms from patients U1, U3, and U5.

Overall, combinations of antibiotics with the antimicrobial peptide exerted synergistic effects in some patients, being more effective than the sum of their activities. However, in other cases, no synergy was seen.

Combinatorial therapies based on conventional antibiotics and antimicrobial peptides could reduce the effective doses of antibiotics, thus minimizing the risk of antibiotic resistance development. We have found meaningful synergistic effects between r(P)ApoB_L_^Ala^ and conventional antibiotics in some extracts. Therefore, we hypothesize that the antimicrobial peptide might perturb the permeability of target bacterial membranes, promoting internalization of the antibiotics and thus potentiating their efficacy [21,23]. Similarly, the increased anti-biofilm activity of the combinations might be due to the peptide solubilizing extracellular polymers, perturbing the biofilm matrix and allowing the antibiotics to diffuse into the biofilm and reach planktonic target cells [23,35]. These findings highlighted that combining r(P)ApoB_L_^Ala^ with antibiotics might prevent infections and the risk of catheter obstruction, crucial for long-term catheterized patients, potentially decreasing antibiotic dosages, thereby reducing the risk of antibiotic use resistance. However, this study has its limitations. First, the lack of generalization due to few samples; second, we could not gain deep insight into any association/causal link between the detected bacteria and UTI pathophysiology; third, the mode of action between antibiotics and Apo-B-derived peptide were not exploited. Today, urologists have to face radical cystectomy complications, including UTIs in the light of antimicrobial resistance [36]. Thereby, patient management requires the knowledge of the patient population, sex, medical history, complicating factors, local antibiotic susceptibility, individual patient characteristics, and predisposing risk factors before the appropriate treatment regimen is chosen [36,37].These results should be taken into account when considering the effectiveness of new antimicrobial strategies in fighting biofilm-based catheter infections. The experiments provide a new insight into the efficacy of novel antimicrobial strategy, i.e., an antimicrobial peptide identified in human apolipoprotein B, alone or in combination with antibiotics, in fighting clinically isolated bacteria from bladder cancer patients after radical cystectomy.

In the age of antibiotic resistance, these data contribute to shedding light on new antimicrobial strategies endowed with antibacterial and antibiofilm activities useful in patients with suspected CAUTIs. Therefore, it is not necessary to treat all patients. On the other hand, patients undergoing radical cystectomy with UCS in about 50% are considered frail and immunocompromised [38]. These patient groups have to be considered individually and the benefit of screening and treatment of ABU should be reviewed in each case [9].

Therefore, even if all patients with incontinent urinary drainage type UCS do not require drug treatment, there will be a fragile and immunocompromised population in which the results obtained from this article could be useful.

However, it is beyond the scope of this study to assess antimicrobial prohylaxis due to the lack of randomized data.

Although there are still many challenges toward clinical applications of AMPs, some of them, such as nisin, gramicidin, polymyxins, daptomycin, and melittin, have been approved by the FDA [39]. In recent years, AMPs have gained huge interest in various fields. Regarding ApoB derived peptides, we have recently demonstrated their great potentiality in industrial and clinical applications. For instance, chitosan functionalized with r(P)ApoB_L_^Pro^ peptide was found to be a promising active coating able to prevent microbial contaminations of chicken meat samples [27]. ApoB-derived peptides were also fund to be antifungal agents [26] and to exert antimicrobial and antibiofilm activities against bacterial strains clinically isolated from cystic fibrosis patients [24] Recently, polydimethylsiloxane (PDMS) was loaded with r(P)ApoB_L_^Pro^ peptide, and the obtained functionalized material was found to be stable, antimicrobial, and biocompatible, thus highlighting the applicability of ApoB-derived peptides in the functionalization of the surfaces of medical devices [40]. To ensure translatability of these molecules, recently, a protease resistant retro-inverso variant of the lead ApoB derive peptide was also synthesized and found to show anti-infective activity in a preclinical mouse model [41]. Of course, future studies will be necessary to implement the findings here presented by using a higher number of clinically isolated catheter communities and by deeply exploring the clinical potentialities of ApoB-derived peptides in combination with the most effective antibiotics.

## 5. Conclusions

The long-term use of biomedical devices represents a severe threat of urology for patients who have undergone radical cystectomy for muscle-invasive bladder cancer. For the management of these patients, it is essential to avoid the risk of severe biofilm-based infections that could lead to indwelling ureteral catheter occlusions, and, in concomitance, to the development of antibiotic resistance strains. In the era of antibiotic resistance, it is urgent to prevent the risk of development of novel multi-drug-resistant strains and the overuse and misuse of antibiotics to keep this weapon sharp. It is critical to evaluate novel, effective antimicrobial strategies for preventing catheter-associated urinary tract infections (CAUTI) difficult to care and the most common nosocomial infections. In conclusion, in the present study, biofilm communities were isolated and characterized, starting from indwelling ureteral catheters. Moreover, it has been demonstrated that biofilm susceptibility to antibiotics and to r(P)ApoB_L_^Ala^ peptide with synergistic/additive effects in at least some cases. Although our findings deserve further investigations to determine their general applicability, this pilot study indicates a novel research perspective in CAUTI prevention and treatment. Future studies will evaluate the possibility of pre-treating ureteral catheters with a combination of antibiotics and antimicrobial peptide to prevent CAUTI.

## Figures and Tables

**Table 1:** Minimum inhibitory concentration (MIC) assays.

	Compound (mg/mL)					
Catheter Extracts	Fosfomycin	Ceftriaxone	Cyprofloxacin	Tetracyclin	Gentamycin	r(P)ApoB_L_^Ala^
U1	0.15–0.3	2.5	0.625 (MIC_90_)	1	1	>0.4
U2	0.15–0.3	2.5	0.625 (MIC_90_)	2 (MIC_90_)	2 (MIC_90_)	>0.4
U3	0.6	0.3–0.6	0.625 (MIC_90_)	2 (MIC_90_)	2 (MIC_90_)	>0.4
U4	0.3–0.6	2.5	0.625 (MIC_90_)	2 (MIC_90_)	2 (MIC_90_)	>0.4
U5	0.15–0.3	2.5	0.625 (MIC_90_)	2 (MIC_90_)	2 (MIC_90_)	>0.4
U6	0.6	0.3–0.6	0.625 (MIC_90_)	2	2 (MIC_90_)	>0.4

MIC_90_: Inhibition of 90% of bacteria.

## Data Availability

We included all the supporting information within the manuscript’s Supplementary Materials. Further information will be provided upon request.

## Supplementary Materials

The following are available online at https://www.mdpi.com/article/10.3390/life12060802/s1, Table S1: Clinical pathological characteristics of a patient whose ureteral catheter bacterial communities were analyzed in the present study. Table S2: Pairwise comparisons using Fisher’s exact test based on the FDR method. Statistical significance threshold ≤0.05. Raw data Fisher’s test on all 6 extracts (three layers) with peptide alone or in combination with fosfomycin or ceftriaxone. Table S3: Pairwise comparisons using Fisher’s exact test based on the FDR method. Statistical significance threshold ≤0.05. Raw data Fisher’s test on all 3 extracts (three layers) with peptide alone or in combination with ciprofloxacin, gentamicin, and tetracycline. Figure S1: CLSM data before (raw) and after outlier removal for U1, U2, U3, U4, U5, and U6 extracts. (A) Raw data refer to the following experimental conditions from the left to the right side: untreated, r(P)ApoB_L_^Ala^, ceftriaxone, fosfomycin, ceftriaxone plus r(P)ApoB_L_^Ala^, fosfomycin plus r(P)ApoB_L_^Ala^. (B) Pairwise comparisons using the “untreated” biofilm as a reference group, Wilcoxon test. Statistically significant difference levels * *p* < 0.05, ** *p* < 0.01, *** *p* < 0.001, **** *p* < 0.0001, n.s. not significant. Figure S2: CLSM data before (raw) and after outlier removal for U1, U3, and U5 extracts. (A) Raw data for each biofilm experimental condition of growth from left to the right side are as follows: untreated, r(P)ApoB_L_^Ala^, ciprofloxacin, gentamycin, tetracycline, ciprofloxacin_ r(P)ApoB_L_^Ala^, gentamycin_ r(P)ApoB_L_^Ala^, tetracycline_ r(P)ApoB_L_^Ala^. (B) Pairwise comparisons using the “untreated” biofilm as a reference group, Wilcoxon test. Statistically significant difference levels * *p* < 0.05, ** *p* < 0.01, *** *p* < 0.001, **** *p* < 0.0001, n.s. not significant. Table S4: Biofilm thickness data and means pairwise comparison of U1, U4, and U5 catheter extracts upon incubation with r(P)ApoB_L_^Ala^, ceftriaxone, and fosfomycin (ANOVA and Tukey’s HSD post hoc test). Table S5: Biofilm thickness data and means pairwise comparison of U1, U3, and U5 catheter extracts upon incubation with r(P)ApoB_L_^Ala^, gentamicin, ciprofloxacin, and tetracycline (ANOVA and Tukey’s HSD post hoc test).
