# Supplementary material for: Novel Antimicrobial Strategies to Prevent Biofilm Infections in Catheters after Radical Cystectomy: A Pilot Study
Source: Life (Basel). 2022 May 27;12(6):802. doi: 10.3390/life12060802 (PMC9225455; doi:10.3390/life12060802)
Supplement: Supplementary file 1 [file life-12-00802-s001.zip › life-1721526-supplementary.pdf]

Supplementary Materials

Table S1. Clinical pathological characteristics of patients whose ureteral catheters bacterial communities have been analysed in the present study.

Patient	U1	U2	U3	U4	U5	U6
**Sex**	M	M	M	M	M	M
**Age (years)**	77	78	76	74	76	72
**Tumor stage[1]**	pT3bN0M0	pT3aN0M0	pT2bN0M0	pT3bN0M0	pT3aN0M0	pT2bN0M0
**Smoking history**	No	Yes	Yes	No	No	Yes
**Previous antibiotics history**	Cephalosporins	Cephalosporins	Cephalosporins	Cephalosporins	Cephalosporins	Cephalosporins
**Diabetes mellitus**	Yes	No	No	Yes	No	No
**Insulin dependence**	Yes	No	No	No	No	No
**Thyroid disease**	No	No	No	Yes	No	No
**Cardiovascular disease**	Yes	Yes	Yes	No	No	No
**Constipation**	Yes	Yes	No	No	No	No
**Recurrent UTI**	Yes	Yes	No	No	No	No
**UTI-associated with fever**	No	Yes	No	No	No	No
**Prostatic tumor at surgery**	Yes	No	No	Yes	No	No

[1]TNB stage system in accordance with European Association of Urology clinical guidelines.

Table S2. Pairwise comparisons using Fisher's exact test based on FDR method. Statistical significance threshold ≤ 0.05. Raw data Fisher's test on all 6 extracts (three layers) with peptide alone or in combination with fosfomycin or ceftriaxone.

	PATIENT U1			PATIENT U2			PATIENT U3			PATIENT U4			PATIENT U5			PATIENT U6		
**Comparisons**	**A:B**	**A:C**	**B:C**	**A:B**	**A:C**	**B:C**	**A:B**	**A:C**	**B:C**	**A:B**	**A:C**	**B:C**	**A:B**	**A:C**	**B:C**	**A:B**	**A:C**	**B:C**
Layers: untreated.biofilm	1	0.188518	0.193921	1	1	1	0.203784	0.07152	0.77209	1	1	1	0.32513	0.43126	1	0.623842	0.155095	0.677943
Layers: r(P)ApoB$_L^{Ala}$	1	1	0.747672	1	0.706923	1	0.115319	0.107477	1	1	1	1	0.675634	0.409719	0.713167	0.626476	0.091036	0.407694
Layers: fosfomycin	0.347861396	0.435139	1	1	0.762779	0.801806	1	0.629576	0.7295	1	1	1	0.664179	0.099924	0.448911	0.623842	0.115315	0.447814
Layers: ceftriaxone	1	0.659107	0.747672	1	0.458675	0.462727	1	0.436841	0.237464	1	0.680584	1	1	0.43126	0.713167	1	0.677943	0.416048
Layers: r(P)ApoB$_L^{Ala}$.F	1	0.752879	1	1	0.767198	1	1	1	1	1	1	0.533427	1	0.448911	0.713167	0.338768	0.041176	0.407694
Layers: r(P)ApoB$_L^{Ala}$.C	1	0.722768	1	0.198779	0.010059	0.233957	0.684485	0.629576	0.194335	1	0.491123	0.853144	1	0.713167	0.455141	0.603037	1	0.407694

A:B Down vs Middle, A:C Down vs Upper, B:C Middle vs Upper layers

r(P)ApoB$_L^{Ala}$.F and r(P)ApoB$_L^{Ala}$.C means combination of antimicrobial peptide and antibiotic fosfomycin or ceftriaxone (1:20 ratio), respectively.

Table S3. Pairwise comparisons using Fisher's exact test based on FDR method. Statistical significance threshold ≤ 0.05. Raw data Fisher's test on all 3 extract (three layers) with peptide alone or in combination with ciprofloxacin, gentamicin, tetracycline.

	**PATIENT U1**			**PATIENT U3**			**PATIENT U5**		
**Comparisons**	A:B	A:C	B:C	A:B	A:C	B:C	A:B	A:C	**B:C**
Layers:untreated.biofilm	0.713481281	1	0.41010959	0.699587897	0.41413788	0.720987	1	0.38524797	0.40604265
Layers:r.P.ApoBL.Ala.	1	0.73455403	1	0.699587897	0.6995879	1	1	1	0.73573222
Layers:ciprofloxacin	1	0.39505294	0.48774828	1	0.6995879	0.71833928	1	1	0.73573222
Layers:gentamicin.	1	0.72586413	0.75993775	1	0.19289257	0.22633812	1	0.72821295	0.22081779
Layers:tetracycline.	0.679343627	0.43322187	1	1	0.7023648	0.43707902	0.349281474	0.06150715	0.43367195
Layers:r.P.ApoBL.Ala.C	1	1	1	1	0.41413788	0.41413788	1	0.72598718	0.7388349
Layers:r.P.ApoBL.Ala.G	1	1	1	0.463509659	1	0.1896386	1	0.17100825	0.06119944
Layers:r.P.ApoBL.Ala.T	0.725864126	0.40417759	0.75035288	1	0.6995879	0.71476204	0.60889869	1	0.73573222

A:B Down vs Middle, A:C Down vs Upper, B:C Middle vs Upper layers

r(P)ApoB$_L^{Ala}$.C and r(P)ApoB$_L^{Ala}$.G and r(P)ApoB$_L^{Ala}$.T means combination of antimicrobial peptide and antibiotic ciprofloxacin or gentamicin or tetracyclin (1:20 ratio), respectively

A

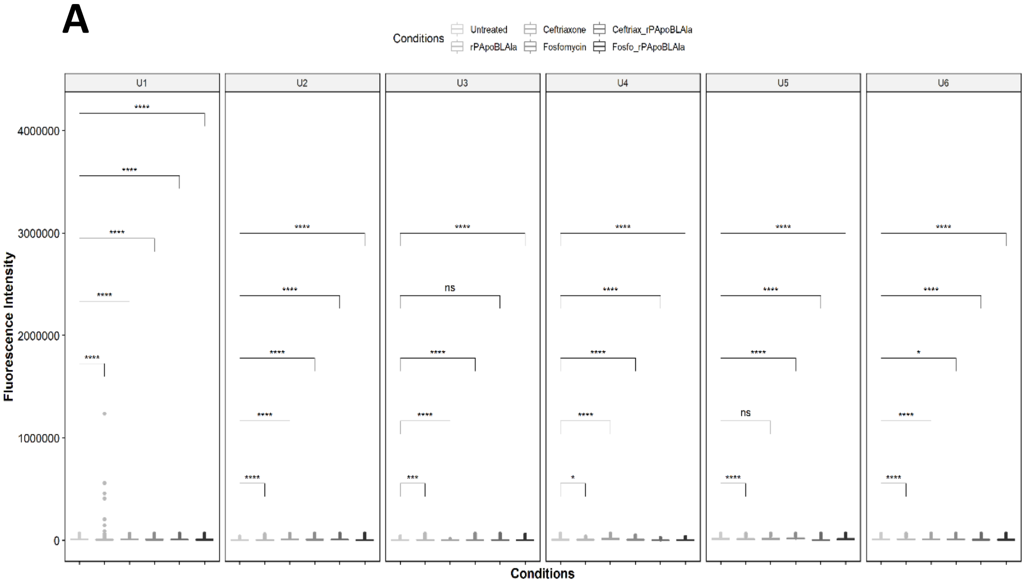

B

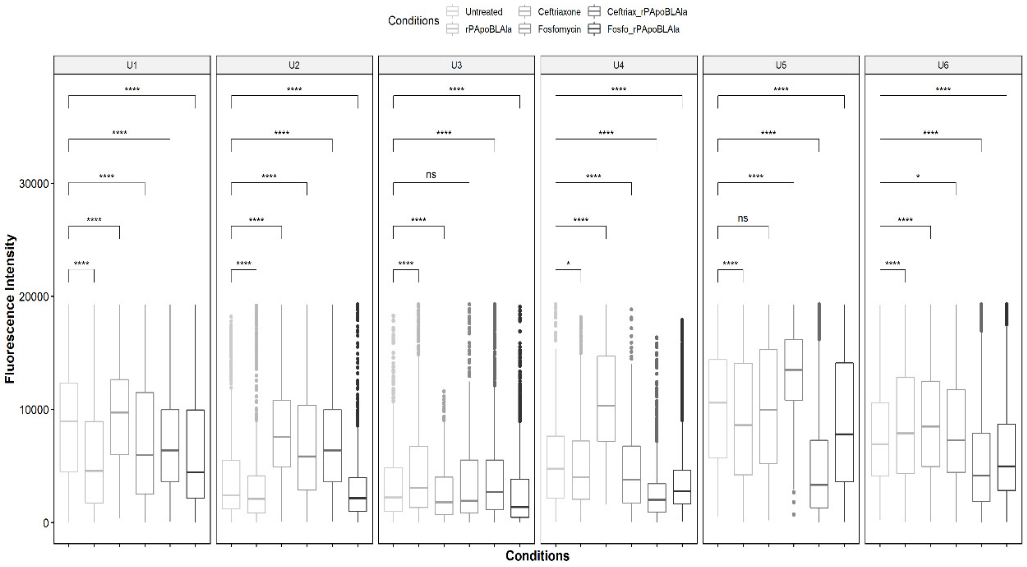

Figure S1: CLSM data before (raw) and after outlier removal for U1, U2, U3, U4, U5, U6 extracts, respectively. A) Raw data refer to the following experimental conditions from the left to the right side: untreated, r(P)ApoB$_L^{Ala}$, ceftriaxone, fosfomycin, ceftriaxone plus r(P)ApoB$_L^{Ala}$, fosfomycin plus r(P)ApoB$_L^{Ala}$. **B)** Pairwise comparisons using the "Untreated" biofilm as reference group, Wilcoxon test. Statistically significant difference levels * $p<0.05$, ** $p<0.01$, ***$p<0.001$, ****$p<0.0001$, n.s. not significant.

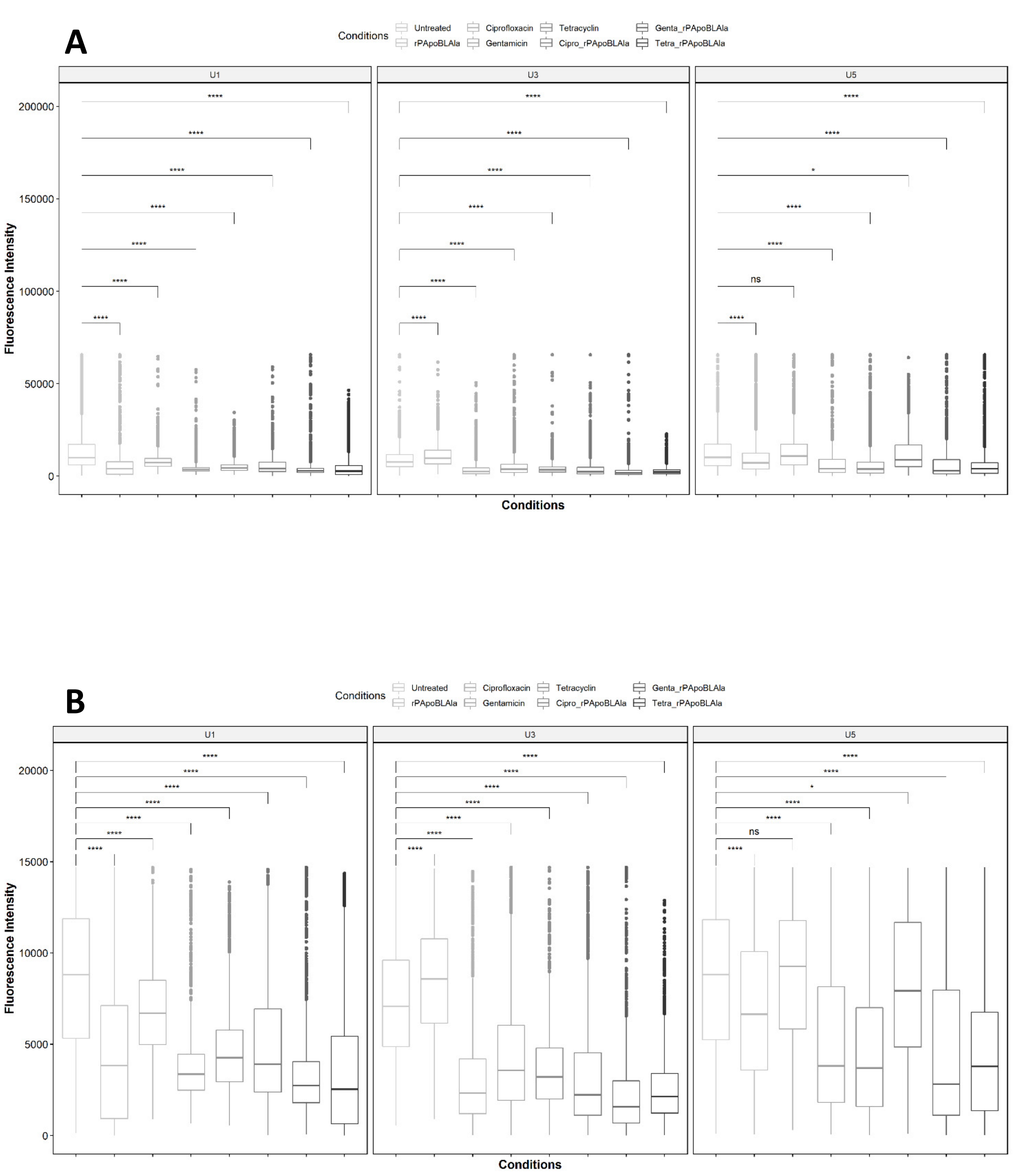

Figure S2: CLSM data before (raw) and after outlier removal for U1, U3, U5 extracts, respectively. A) Raw data for each biofilm experimental condition of growth from left to the right side are as follow: untreated, r(P)ApoBLAla, ciprofloxacin, gentamycin, tetracycline, ciprofloxacin_r(P)ApoBLAla, gentamycin_r(P)ApoBLAla , tetracycline_r(P)ApoBLAla. **B)** Pairwise comparisons using the "untreated" biofilm as reference group, Wilcoxon test. Statistically significant difference levels * $p<0.05$, ** $p<0.01$, ***$p<0.001$, ****$p<0.0001$, n.s. not significant.

Table S4. Biofilm thickness data and means pairwise comparison of U1, U4 and U5 catheter extracts upon incubation with r(P)ApoBLAla, ceftriaxone and fosfomycin (Anova and Tukey HSD post hoc test).

**Incubation variable**	**Ceftriaxone**	**Fosfomycin**	**r(P)ApoBL Ala**	**Ceftriaxone:r(P)ApoB$_L^{Ala}$**	**Fosfomycin:r(P)ApoB$_L^{Ala}$**
**Untreated**	ns	ns	ns	0.01638	ns
**Ceftriaxone**		ns	ns	ns	ns
**Fosfomycin**	ns		ns	ns	ns
**r(P)ApoBL Ala**	0.033484	ns		0.005676	ns
**Ceftriaxone:r(P)ApoB$_L^{Ala}$**	ns	ns	ns		ns
**Fosfomycin:r(P)ApoB$_L^{Ala}$**	ns	ns	ns	ns	

ns: not significant (p adjusted <0.05).

Table S5. Biofilm thickness data and means pairwise comparison of U1, U3 and U5 catheter extracts upon incubation with r(P)ApoBLAla, gentamicin, ciprofloxacin and tetracycline (Anova and Tukey HSD post hoc test).

**Incubation variable**	**Ciprofloxacin**	**Gentamicin**	**Tetracycline**	**r(P)ApoBL Ala**	**Ciprofloxacin:r(P) ApoB$_L^{Ala}$**	**Gentamicin:r(P)Ap oB$_L^{Ala}$**	**Tetracycline:r(P)A poB$_L^{Ala}$**
**Untreated**	ns	1.8038E-05	2.2433E-02	2.2433E-02	8.9938E-04	7.4429E-06	7.4429E-06
**Ciprofloxacin**		7.6266E-03	ns	ns	ns	2.5949E-03	2.5949E-03
**Gentamicin**	7.6266E-03		2.2433E-02	2.2433E-02	ns	ns	ns
**Tetracycline**	ns	2.2433E-02		ns	ns	ns	7.6266E-03
**r(P)ApoBL Ala**	ns	2.2433E-02	ns		ns	7.6266E-03	7.6266E-03
**Ciprofloxacin:r(P)ApoB$_L^{Ala}$**	ns	ns	ns	ns		ns	ns
**Gentamicin:r(P)ApoB$_L^{Ala}$**	2.5949E-03	ns	ns	7.6266E-03	ns		ns
**Tetracycline:r(P)ApoB$_L^{Ala}$**	2.5949E-03	ns	7.6266E-03	7.6266E-03	ns	ns	

ns: not significant (p adjusted <0.05).
